# Zuranolone as a Novel Therapy for Postpartum Depression: A Narrative Review of Current Evidence and Future Directions

**DOI:** 10.7759/cureus.101038

**Published:** 2026-01-07

**Authors:** Israa Elkashif, Sindhu Vithayathil, Maryam Walizada, Gowtham Siddi, Srirachana Reddy Gumireddy, Mariette Anto, Ann Kashmer Yu

**Affiliations:** 1 Psychiatry, Royal College of Surgeons in Ireland, Dublin, IRL; 2 General Medicine, American University of Antigua, Coolidge, ATG; 3 Internal Medicine, Mary Washington Hospital, Fredericksburg, USA; 4 Medicine, NRI Academy of Medical Sciences, Guntur, IND; 5 Medicine, Apollo Institute of Medical Sciences and Research, Hyderabad, IND; 6 Orthopaedics, King's Mill Hospital, Sutton-in-Ashfield, GBR; 7 Internal Medicine, California Institute of Behavioral Neurosciences & Psychology, Fairfield, USA

**Keywords:** gaba, neuroactive steroid, perinatal depression, postpartum depression, zuranolone

## Abstract

Postpartum depression (PPD) is a debilitating complication of childbirth and is associated with significant consequences for maternal well-being and infant development. Current pharmacological treatments have many limitations, including a delayed onset of action, variable response rates, and concerns about tolerability, underscoring the need for novel therapeutic approaches. Zuranolone has recently emerged as a novel oral therapy specifically for the treatment of PPD and represents a potential turning point in the management of PPD. This narrative review outlines the evolving evidence base for zuranolone, including current understanding of its efficacy and tolerability, and explores practical considerations for its use. The potential role of zuranolone within the broader treatment landscape is discussed, alongside key uncertainties regarding duration of effect, longer-term outcomes and accessibility. By synthesising available literature and identifying key knowledge gaps, this review aims to clarify the evolving role of zuranolone in the treatment of PPD, while highlighting priorities for future research and clinical practice.

## Introduction and background

Postpartum depression

Postpartum depression (PPD) is a global health crisis and is one of the most common complications of childbirth. It affects approximately one in seven women during pregnancy or within one year following childbirth, with suicide being the second most common cause of mortality for women postpartum [1]. The DSM-5-TR does not identify PPD as a distinct disorder; rather, it classifies it as an episode of major depressive disorder (MDD) occurring during pregnancy or within four weeks of delivery [1]. PPD is characterised by depressive symptoms, including pervasive low mood, anhedonia, loss of appetite, sleep disturbance, fatigue, feelings of hopelessness and helplessness, and difficulty concentrating, in addition to difficulty bonding with the baby, feelings of humiliation, self-blame, and hostility towards the baby. Additionally, women diagnosed with PPD exhibit a higher likelihood of engaging in suicidal behaviour when compared to women in the same population or their full sisters who do not have this diagnosis. Moreover, this increased risk was observed in women both with and without prior psychiatric history, indicating that PPD contributes additional risk for suicidal behaviour beyond that linked to earlier psychiatric history [2]. The risk of suicidal behaviour has been found to be highest in the immediate period following diagnosis of PPD and, in some cases, although attenuated, may potentially last up to 18 years [2]. Despite this knowledge, PPD remains underdiagnosed and undertreated [3].

Adverse consequences of untreated PPD

Not only does PPD pose significant adverse consequences for the mother, including mental, physical, and social, but infants of depressed mothers also manifest lower social engagement, more negative emotions, and experience higher cortisol reactivity [4]. There is also an association between PPD and poor language and cognitive development in children [5]. Women with PPD may also experience thoughts of harming their child, which can manifest in the form of plans and intent to harm the child, or obsessional thoughts in which the mother is highly distressed by the thoughts or images of harming her child and has no intent to act on them [5]. Left untreated, PPD is likely to result in recurring episodes of depression impacting the mother, her child, and her family at large [5]. Children of women with persistent (over two months) and severe depression are at an increased risk of displaying behavioural problems by age 3.5, lower mathematical grades, and depression in adolescence when compared with children of women with PPD that did not persist beyond two months [6]. Women with persistent PPD may continue experiencing depressive symptoms for at least 11 years after giving birth [6], thus highlighting the need for urgent identification of women at risk of developing PPD and their prompt treatment.

Pathophysiology of PPD

The exact pathophysiology of PPD is complex and not yet fully understood; however, there is evidence that it is multifactorial, involving biological and hormonal factors, genetics, and immune function. Some studies have noted that there appears to be a subgroup of women who are particularly susceptible to hormonal shifts across their lifespan and that these shifts are capable of provoking affective dysregulation in women who have a genetic predisposition [5]. Twin studies also suggest a genetic element to PPD, with the serotonin (5-HTTLPR) and oxytocin (OXTR) genes having the most significant influence [5,6]. Furthermore, alternative theories examine changes in the neuroendocrine system that disrupt gamma-aminobutyric acid (GABA) signalling and lead to decreased levels of Allopregnanolone [7-9]. The complex and poorly understood pathophysiology of PPD not only poses challenges for treatment but also presents opportunities for researchers to explore novel therapeutic approaches.

Barriers to care

Despite the plethora of treatment options available, PPD is commonly overlooked in clinical settings [3,10]. This can be attributed to multiple reasons. One major obstacle is the stigma surrounding mental illness, including the fear of being perceived as a "bad mother". The stigma is often more pronounced amongst ethnic minority groups, influenced by cultural beliefs and identity [10]. Additional contributing factors include insufficient postgraduate training for healthcare professionals in postpartum mental health, the absence of national guidelines for the prevention, treatment, and management of PPD, and the lack of well-structured perinatal mental health services. Further barriers include unclear referral pathways, a shortage of specialists, long waiting periods, and financial constraints [10].

Current treatments

Psychological interventions, including cognitive behavioural therapy and interpersonal therapy, are the first-line treatment strategies for mild to moderate PPD and have been shown to provide benefits both acutely following treatment and in longer-term follow-up (six months after completion of treatment) [5]. For more severe cases, or when psychological interventions are insufficiently effective, antidepressant medication may be required, with selective serotonin reuptake inhibitors (SSRIs) being the first-line drug of choice [5]. Although most antidepressants are not contraindicated during breastfeeding, and no single antidepressant has been clearly shown to be superior, SSRIs tend to be used more frequently due to the lower levels of infant exposure through lactation [5]. The American College of Obstetricians and Gynaecologists (ACOG) recommends the use of SSRIs, selective norepinephrine reuptake inhibitors (SNRIs), and tricyclic antidepressants (TCAs) in the treatment of PPD [11]. However, caution must be exercised. Some studies have indicated a possible link between SNRIs and an increased risk of preeclampsia and miscarriage [11,12]. The UK's Specialist Pharmacy Service (SPS) provides cautionary guidance regarding the use of TCAs, stating that although most TCAs can be safely used in pregnancy, they should be used with caution. Although current evidence shows that levels in breastmilk are very low, there is a possibility of the infant developing neonatal withdrawal syndrome, poor feeding, urinary retention, and sedation (when exposed through breastmilk). They advise that imipramine and clomipramine are the preferred choices, but that there is no need to change a TCA used successfully in pregnancy so long as the infant is healthy and born at full term [13].

Traditional antidepressants are characterised by a slow onset of action, posing a significant challenge in treating PPD, given the potential for rapid deterioration and lasting effects on both the mother and her infant if not treated promptly [14]. The European Medicines Agency states that conventional antidepressants typically require four to six weeks to show clinical improvement [14]. This improvement may be further delayed if the patient does not respond to treatment or deteriorates, necessitating a switch to an alternative antidepressant. This delay can be detrimental to women in the postpartum period, as this time is crucial for forming an emotional bond with the newborn child and managing new responsibilities and roles within the family unit. Although it is recommended that antidepressants are continued for a duration of at least six months, medication adherence amongst women with PPD tends to be poor, with one nationwide cohort study revealing that a significant proportion of women only filled one prescription, and that less than 28% remained on treatment at one-year follow-up [15]. A recent systematic review found suboptimal medication adherence in 46-83% of patients, and that adverse effects, in particular somnolence, were associated with non-adherence and early discontinuation [16]. Given that only 30.8% of cases are identified in clinical settings, with only 15.8% actually receiving treatment and only 3.2% achieving remission [17], it is paramount to improve adherence support strategies in order to optimize treatment outcomes and reduce the burden of untreated illness.

Novel therapies

In the United States, the Food and Drug Administration (FDA) has not approved serotonergic or other traditional antidepressants for PPD, thus making their use in this context off-label. The majority of randomised control trials (RCTs) have evaluated selective serotonin reuptake inhibitors (SSRIs); however, the evidence suggesting their superiority over placebo in the treatment of PPD is of low certainty [18]. The use of traditional antidepressants is largely based on clinical experience rather than solid scientific evidence. The recent emergence of gamma-aminobutyric acid-ergic (GABAergic) neuroactive steroids, such as Brexanolone and Zuranolone, represents a significant therapeutic advance for the targeted treatment of PPD [17]. The medications offer the potential for a more rapid therapeutic response and remission compared to conventional antidepressants. Zuranolone, a GABAergic neuroactive steroid, was approved by the FDA in 2023 as the first-ever oral treatment for PPD [19]. It is similar to its predecessor, Brexanolone, the first drug approved specifically for PPD [20]. Unlike Brexanolone, it is available in oral form and does not require administration under the FDA's Risk Evaluation and Mitigation Strategy (REMS) program [20]. Zuranolone offers a novel therapeutic approach with enhanced accessibility, presenting an exciting option for treating women with PPD. This narrative review seeks to examine the current literature on zuranolone as a novel therapeutic option for PPD and to evaluate its potential role in contemporary treatment paradigms.

## Review

Pharmacology

GABA is the primary inhibitory neurotransmitter in the central nervous system (CNS) and plays a crucial role in maintaining balanced neuronal activity throughout the brain [20]. Zuranolone is a neuroactive steroid that modulates GABAA receptors, acting similarly to the endogenous compound allopregnanolone. Its mechanism of action is believed to involve positive allosteric modulation of GABAA receptors, resulting in increased receptor expression and amplified GABAA-mediated signalling. Unlike benzodiazepines, which can only modulate GABAA receptors that contain γ subunits, neuroactive steroids like zuranolone can modulate both synaptic and extrasynaptic receptors, including those with δ subunits [20]. This broader action may contribute to more rapid and sustained inhibitory effects, which translate into enhanced mood stabilisation [21].

Neuroactive steroids play a vital role in regulating the hypothalamic-pituitary-adrenal (HPA) axis, both at baseline and under acute and chronic stress conditions. Beyond their involvement in HPA axis regulation, neuroactive steroids exhibit anti-inflammatory and neurotrophic properties within the CNS [18]. Extensive research suggests that PPD is linked to altered functioning of the stress hormone system, along with distinct vulnerabilities within the serotonergic and GABAergic pathways [18].

Pharmacokinetics

Zuranolone is administered orally once daily in the evening for a duration of 14 days, typically at a dose of 50mg. It should be taken with a high-fat meal (400-1000 calories, 25-50% fat) to optimise absorption. Zuranolone is highly metabolised by cytochrome P450 3A4 (CYP3A4); therefore, concomitant use with CYP3A4 inducers should be avoided, and the dosage should be reduced if used concomitantly with a potent CYP3A4 inhibitor [22].

Key clinical trials

ROBIN Study

This trial, conducted by Deligiannidis et al., aimed to demonstrate the safety and efficacy of zuranolone in the treatment of PPD [23]. It was a phase 3, double-blind, randomised, placebo-controlled trial involving 151 women with a diagnosis of PPD. Participants who received 30mg of zuranolone daily for 14 days showed a statistically significant reduction in depressive symptoms compared to those receiving a placebo, as assessed by the 17-item Hamilton Rating scale for depression. Participants receiving zuranolone also experienced rapid and sustained improvements in anxiety and improved global and maternal functioning. zuranolone was generally well-tolerated, with the most common adverse events being somnolence (11%), nausea (8%), dizziness (6%), vomiting (6%), abnormal dreams (6%), and hyperhidrosis (6%) [22]. Due to the follow-up ending at day 45, the long-term efficacy and safety of zuranolone cannot be determined from this study [23].

SKYLARK Study

This study, also conducted by Deligiannidis et al., aimed to evaluate the safety and efficacy of zuranolone in women with severe PPD [24]. It was a double-blind, phase 3, placebo-controlled, parallel group study involving 196 women diagnosed with severe PPD. Those who received 50mg of zuranolone daily demonstrated statistically significant improvements in their depressive symptoms compared to those taking a placebo. Significant symptom improvements were also observed on days 3, 28, and 45. Zuranolone was generally well tolerated, with the most commonly reported adverse events being somnolence (7.1%), dizziness (6.1%), and sedation (3.1%) [24].

Supporting analyses and reviews

Zhang et al. conducted a systematic review and meta-analysis to evaluate the efficacy and tolerability of different pharmacotherapies for PPD [25]. Based on the cumulative ranking curve (SUCRA) analysis, estradiol, paroxetine, and zuranolone ranked higher in efficacy than other antidepressants. Regarding the mean change in HAMD-17 scores, only estradiol and brexanolone demonstrated statistically significant superiority over placebo. However, brexanolone was shown to be less well-tolerated than other antidepressants [25].

Giannopoulos et al. conducted an in-depth review of the clinical utility of zuranolone and discussed the key aspects of clinical decision-making in patients with PPD [18]. They recommend that oral zuranolone or IV brexanolone be used as the first-line treatment in women with moderate to severe unipolar PPD, given their rapid onset and acute treatment course. Zuranolone has demonstrated clinical efficacy, including improvements in anxiety, insomnia, and some aspects of health-related quality of life, when administered in women with PPD up to 12 months postpartum [18].

Raja et al. conducted a rigorous systematic review and meta-analysis to evaluate the safety and efficacy of zuranolone in the treatment of MDD and PPD, with or without concurrent insomnia [26]. Their primary outcome was the change in HAMD scores, while the secondary outcomes included the Montgomery-Åsberg Depression Rating Scale, the Hamilton Anxiety Scale, the Bech Scale, as well as the occurrence of treatment-related side effects and serious adverse events. Although their outcomes favoured the use of zuranolone, these particular outcomes did not achieve statistical significance. Nonetheless, the overall findings supported the use of the drug. They advise interpreting these results with caution and highlight the need for further studies to confirm and strengthen these conclusions [26].

Winslow et al. conducted a systematic review and meta-analysis to assess the efficacy of zuranolone in the treatment of MDD and PPD [27]. They found that a once-daily, 14-day oral course of zuranolone causes a clinically significant and sustained (at day 45) improvement in symptoms of PPD and in maternal functioning; however, the improvement was not significantly sustained beyond the 15-day period for those with MDD. Their study also revealed that neither men nor women experienced an increase in sexual dysfunction from baseline when taking 30mg of zuranolone, a significant finding given that one in six women experiences some degree of sexual dysfunction when taking conventional antidepressants, which may contribute to the issue of non-adherence with medication [27].

Lactation

Medications with a relative infant dose (RID), defined as the percentage of maternal weight-adjusted dose received by the infant via breast milk, of less than 10% are typically regarded as safe for use during breastfeeding (although this is not the only factor to be considered when evaluating the safety of medication use during lactation) [28]. A phase 1 open-label study by Deligiannidis et al. assessed the extent of zuranolone transfer to breast milk in healthy, non-pregnant, lactating women [28]. They estimated that the RID associated with once-daily dosing of zuranolone 50mg for 14 days is 0.74% and 0.98% for milk intakes of 150 mL/kg and 200 mL/kg per day, respectively. These values are similar to or less than the RID values for commonly prescribed antidepressants. ACOG endorses a shared decision-making approach when considering treatment with zuranolone, weighing the risks and benefits of treatment and the advantages of breastfeeding for infant health and development [18,28].

Safety

The Shoreline study is an ongoing Phase 3, open-label, longitudinal clinical trial that evaluates the long-term safety and tolerability of zuranolone, as well as the need for repeat dosing, in patients with MDD [29]. Participants received an initial 14-day course of zuranolone (30mg or 50mg) and were followed for up to one year within the study, with five repeat treatment courses permitted over the 12 month period. The interim results reveal that the most treatment-emergent adverse events (TEAEs) are mild to moderate in severity, with the most common being somnolence, dizziness, headache, and sedation, consistent with previous studies [18,22-25]. 4.4% of those receiving 30mg and 8% of those receiving 50mg withdrew from the study due to TEAEs. There was no increase in suicidal ideation or behaviour compared to baseline amongst those who received zuranolone. Zuranolone demonstrated a consistent safety profile across repeated treatment courses, with significant variation in the incidence or type of TEAE when compared with the initial treatment course [29]. It should be noted, however, that the study population comprised patients with MDD rather than PPD, limiting the generalizability of its findings.

Lin et al. conducted a meta-analysis evaluating the efficacy and safety of zuranolone in MDD [30]. TEAEs were found to be consistent with previous studies, including somnolence, dizziness, sedation and headache. The incidence of TEAEs increasing with higher dosing schedules [30]. Treatment and placebo groups had comparable drop-out rates, suggesting that zuranolone is generally well-tolerated. They discuss that in a study of patients with MDD, participants randomised to zuranolone (1%) experienced suicidal ideation or behaviour more frequently than patients receiving placebo (0%), and that this imbalance has not been noted in studies of PPD. They posit that this finding may have led to the FDA's decision to prioritise approving zuranolone for the treatment of PPD rather than MDD [30].

Supplementary clinical evidence

Although substantial research over recent decades has advanced our understanding of PPD, the experience of anxiety during pregnancy and the postpartum period remains comparatively underexplored [31]. Not only are anxiety disorders more prevalent than depression in the postpartum period, but PPD is also characterised by a higher incidence of anxiety symptoms compared to depressive episodes unrelated to pregnancy. Some studies report a 20-25% prevalence of anxiety disorders during pregnancy and 15-20% in the postpartum period [31]. 75.5% of participants in the SKYLARK study exhibited moderate to severe anxiety, as indicated by a HAM-A score of ≥20 [24]. Treatment with zuranolone was associated with both rapid (by day 3) and sustained improvements in anxiety (by day 45) [23]. Improvements in insomnia symptoms were also observed, in addition to improvements in self-reported functional health [32]. These results hold potential significance for clinical practice as rapid improvements in depression, anxiety, and insomnia may be achieved without a need for polypharmacy and thus reducing the risk for adverse events, drug interactions, and poor adherence [32].

Early recognition of PPD and timely initiation of appropriate, individualised treatment are essential to mitigating both the immediate and long-term consequences for mothers, infants, and families [18,33,34]. Unfortunately, PPD is common but often underdiagnosed and undertreated, with many studies documenting barriers to help-seeking and gaps in care for women [35]. Understanding the barriers to help-seeking across the various layers of the socio-ecological model is crucial for developing a comprehensive strategy to prevent, detect early, and effectively manage perinatal mental health conditions [35].

Although RCTs have demonstrated brexanolone's efficacy, a recent Cochrane review reports that brexanolone may have little to no effect compared to placebo in the treatment of PPD [36]. In contrast, zuranolone has demonstrated rapid and clinically meaningful improvements in depressive symptoms in a number of Phase 3 trials involving adult women within twelve months postpartum. Given its short, two-week treatment course and rapid onset of action, zuranolone may be particularly beneficial for individuals with severe PPD, those with suicidal ideation, and those who have not responded to or cannot tolerate traditional antidepressants.

Somnolence and sedation, among zuranolone’s most commonly reported side effects, may offer therapeutic benefit in addressing insomnia, a frequent comorbidity in patients with PPD [32]. However, its CNS depressant effects warrant caution. A boxed warning from the FDA advises against engaging in potentially hazardous activities, such as driving, for at least 12 hours after administration [37]. Concurrent use with other CNS depressants, such as benzodiazepines, alcohol or opioids, or with drugs that increase the concentration of zuranolone, may increase the risk of sedation, confusion, falls and respiratory depression [22]. Furthermore, zuranolone’s CNS depressant effects may impair alertness and psychomotor function [37], which is relevant for individuals who are primary caregivers of infants. 

Zuranolone has some potential for abuse owing to its dose-dependent adverse reactions, including euphoric mood, feelings of intoxication and sedation. It is controlled as a Schedule IV substance in the US due to its risks of misuse and abuse [38]. Additionally, zuranolone has the potential to cause mild to moderate discontinuation symptoms, including insomnia, palpitations, nightmares, hyperhidrosis and paranoia, suggesting a potential for physiologic dependence [37]. 

There are no human studies on the use of zuranolone during pregnancy, therefore human teratogenic risk is unknown [37]. Based on animal studies, there is some potential for teratogenicity with zuranolone if taken during pregnancy. In rats, increased fetal malformations and embryofetal death were noted, indicating teratogenic potential at sufficiently high exposures in animal models [22]. As evidence in pregnant women is insufficient, its use during pregnancy is not recommended, and effective contraception should be continued for one week after the final dose [37].

Zuranolone is approved by the FDA for the treatment of PPD in adults aged 18 years and older [19]. Most clinical trials have been conducted in adult populations of reproductive age, with pivotal PPD trials enrolling women aged 18-45 [23,24]. Most clinical trials excluded women with bipolar disorder, schizophrenia, or other psychotic disorders, as well as those with active suicidal ideation, recent suicide attempts, substance misuse disorders, or significant medical comorbidities. These exclusion criteria limit the generalizability of findings to the broader population, particularly among more vulnerable or clinically complex patients. Additionally, existing evidence is also limited by the relatively short follow-up duration, typically up to 45 days post-treatment. This leaves significant uncertainty regarding the sustainability of its therapeutic benefits, the risk of relapse, and its long-term safety profile. It is essential to determine whether a two-week course of zuranolone provides durable remission or whether maintenance strategies or repeat courses are necessary.

Although zuranolone is FDA-approved in the US, regulatory approval outside of the US remains pending. Currently, the cost of a 14-day course of zuranolone stands at $15,900 [39]. This cost is significantly higher than the price of generic SSRIs, such as fluoxetine, which may cost as little as $16 per month for uninsured individuals [40]. Sage Therapeutics, the biopharmaceutical company that sells zuranolone, justifies this cost by highlighting a 2017 model that estimates the average cost of untreated perinatal mood and anxiety disorders per mother-child pair to be $32,00 [41]. Therefore, wider adoption of zuranolone may be constrained by cost.

## Conclusions

The advent of neuroactive steroid-based antidepressants, such as zuranolone, marks a turning point in the treatment of PPD. Zuranolone's fast-acting nature and brief treatment duration make it a particularly appealing option in the perinatal setting, where adherence to treatment and timely symptom relief are invaluable for both maternal and infant well-being. Additionally, its clinical profile aligns well with managing the broader range of symptoms that often accompany postnatal mood disorders, including anxiety and insomnia.

Zuranolone has not been studied in pregnant populations, and therefore, individuals who choose not to use contraception may be better candidates for alternative antidepressants. Zuranolone should not be used in pregnant individuals unless the benefits clearly outweigh the risks. The sedative profile of zuranolone warrants careful consideration, particularly when considering treatment in individuals for whom sedation may pose additional risks. In such cases, antidepressants that lack sedative effects may represent a more appropriate therapeutic option. Future research should prioritise large-scale, long-term studies to establish the durability of response, safety profile, and integration of zuranolone across diverse clinical contexts and healthcare systems.

## References

[1] Carlson K, Mughal S, Azhar Y, Siddiqui W (2025). Perinatal depression. StatPearls [Internet].

[2] Yu H, Shen Q, Bränn E, Yang Y, Oberg AS, Valdimarsdóttir UA, Lu D (2024). Perinatal depression and risk of suicidal behavior. JAMA Netw Open.

[3] Frieder A, Fersh M, Hainline R, Deligiannidis KM (2019). Pharmacotherapy of postpartum depression: current approaches and novel drug development. CNS Drugs.

[4] Liu X, Wang S, Wang G (2022). Prevalence and risk factors of postpartum depression in women: a systematic review and meta‑analysis. J Clin Nurs.

[5] Stewart DE, Vigod SN (2019). Postpartum depression: pathophysiology, treatment, and emerging therapeutics. Annu Rev Med.

[6] Chandra JH, Kurniawan C, Puspitasari IM (2024). Genetic markers associated with postpartum depression: a review. Neuropsychiatr Dis Treat.

[7] Suryawanshi O 4th, Pajai S (2022). A comprehensive review on postpartum depression. Cureus.

[8] Maguire J (2019). Neuroactive steroids and GABAergic involvement in the neuroendocrine dysfunction associated with major depressive disorder and postpartum depression. Front Cell Neurosci.

[9] Deligiannidis KM, Fales CL, Kroll-Desrosiers AR (2019). Resting-state functional connectivity, cortical GABA, and neuroactive steroids in peripartum and peripartum depressed women: a functional magnetic resonance imaging and spectroscopy study. Neuropsychopharmacology.

[10] Dominiak M, Antosik-Wojcinska AZ, Baron M, Mierzejewski P, Swiecicki L (2021). Recommendations for the prevention and treatment of postpartum depression. Ginekol Pol.

[11] (2023). Treatment and management of mental health conditions during pregnancy and postpartum: ACOG Clinical Practice Guideline No. 5. Obstet Gynecol.

[12] Viswanathan M, Middleton JC, Stuebe AM (2021). Maternal, fetal, and child outcomes of mental health treatments in women: a meta‑analysis of pharmacotherapy. Psychiatr Res Clin Pract.

[13] (2025). Using tricyclic antidepressants during breastfeeding. http://www.sps.nhs.uk/articles/using-tricyclic-antidepressants-during-breastfeeding..

[14] American Academy of Family Physicians (2025). Guideline on clinical investigation of medicinal products in the treatment of depression. Am Fam Physician.

[15] Rasmussen MH, Strøm M, Wohlfahrt J, Videbech P, Melbye M (2017). Risk, treatment duration, and recurrence risk of postpartum affective disorder in women with no prior psychiatric history: a population-based cohort study. PLoS Med.

[16] Niarchou E, Roberts LH, Naughton BD (2024). What is the impact of antidepressant side effects on medication adherence among adult patients diagnosed with depressive disorder: a systematic review. J Psychopharmacol.

[17] Kaufman Y, Carlini SV, Deligiannidis KM (2022). Advances in pharmacotherapy for postpartum depression: a structured review of standard-of-care antidepressants and novel neuroactive steroid antidepressants. Ther Adv Psychopharmacol.

[18] Giannopoulos A, Singh J, Deligiannidis KM (2025). Clinical utility of zuranolone for postpartum depression: a narrative review. Neuropsychiatr Dis Treat.

[19] (2025). FDA approves first oral treatment for postpartum depression. http://www.fda.gov/news-events/press-announcements/fda-approves-first-oral-treatment-postpartum-depression.

[20] St Onge E, Patel P, Whitner C (2025). Zuranolone for the treatment of postpartum depression. J Pharm Technol.

[21] Cutler AJ, Mattingly GW, Maletic V (2023). Understanding the mechanism of action and clinical effects of neuroactive steroids and GABAergic compounds in major depressive disorder. Transl Psychiatry.

[22] (2025). ZURZUVAE™ (zuranolone) capsules, for oral use. https://www.accessdata.fda.gov/drugsatfda_docs/label/2023/217369s000lbl.pdf.

[23] Deligiannidis KM, Meltzer-Brody S, Gunduz-Bruce H (2021). Effect of zuranolone vs placebo in postpartum depression: a randomized clinical trial. JAMA Psychiatry.

[24] Deligiannidis KM, Meltzer-Brody S, Maximos B (2023). Zuranolone for the treatment of postpartum depression. Am J Psychiatry.

[25] Zhang Q, Dai X, Li W (2022). Comparative efficacy and acceptability of pharmacotherapies for postpartum depression: a systematic review and network meta-analysis. Front Pharmacol.

[26] Raja A, Ahmed S, Basit Ali Siddiqui M (2024). Evaluating the safety and efficacy of zuranolone in the management of major depressive disorder and postpartum depression, with or without concurrent insomnia: a rigorous systematic review and meta-analysis. Front Psychiatry.

[27] Winslow M, White E, Rose SJ, Salzer E, Nemec EC 2nd (2024). The efficacy of zuranolone versus placebo in postpartum depression and major depressive disorder: a systematic review and meta-analysis. Int J Clin Pharm.

[28] Deligiannidis KM, Bullock A, Nandy I (2024). Zuranolone concentrations in the breast milk of healthy, lactating individuals: results from a phase1 open-label study. J Clin Psychopharmacol.

[29] Cutler AJ, Mattingly GW, Kornstein SG (2023). Long-term safety and efficacy of initial and repeat treatment courses with zuranolone in adult patients with major depressive disorder: interim results from the open-label, Phase 3 SHORELINE Study. J Clin Psychiatry.

[30] Lin YW, Tu YK, Hung KC (2023). Efficacy and safety of zuranolone in major depressive disorder: a meta-analysis of factor effect and dose-response analyses. EClinicalMedicine.

[31] Nakić Radoš S, Tadinac M, Herman R (2018). Anxiety during pregnancy and postpartum: course, predictors and comorbidity with postpartum depression. Acta Clin Croat.

[32] Deligiannidis KM, Citrome L, Huang MY (2023). Effect of zuranolone on concurrent anxiety and insomnia symptoms in women with postpartum depression. J Clin Psychiatry.

[33] Barnes KN, Vogl CM, Nelson LA (2024). Zuranolone: the first FDA-approved oral treatment option for postpartum depression. Ann Pharmacother.

[34] Goodman JH (2004). Paternal postpartum depression, its relationship to maternal postpartum depression, and implications for family health. J Adv Nurs.

[35] Place JM, Renbarger K, Van De Griend K, Guinn M, Wheatley C, Holmes O (2024). Barriers to help-seeking for postpartum depression mapped onto the socio-ecological model and recommendations to address barriers. Front Glob Womens Health.

[36] Wilson CA, Robertson L, Ayre K, Hendon JL, Dawson S, Bridges C, Khalifeh H (2025). Brexanolone, zuranolone and related neurosteroid GABA(A) receptor positive allosteric modulators for postnatal depression. Cochrane Database Syst Rev.

[37] (2025). Zuranolone (monograph). https://www.drugs.com/monograph/zuranolone.html.

[38] (2026). Sage Therapeutics announces fourth quarter and full year 2024 financial results and highlights pipeline and business updates. http://www.sec.gov/Archives/edgar/data/1597553/000119312525024319/d933485dex991.htm.

[39] Buntz B (2025). Is the new $15,900 postpartum depression pill worth it?. Drug Discovery & Development.

[40] Dunleavy K (2025). After scoring limited FDA nod, Sage and Biogen call an audible on pricing strategy for Zurzuvae. Fierce Pharma.

[41] (2026). Sage Therapeutics announces third quarter 2023 financial results and highlights pipeline and business progress. https://www.businesswire.com/news/home/20231107571536/en/Sage-Therapeutics-Announces-Third-Quarter-2023-Financial-Results-and-Highlights-Pipeline-and-Business-Progress.

